# Nanoscopy and an extended lateral approach can improve the management of latero-central segments in tibial plateau fractures: a cadaveric study

**DOI:** 10.1007/s00068-022-02188-3

**Published:** 2022-12-09

**Authors:** Peter Behrendt, M. T. Berninger, G. Thürig, J. Dehoust, J. Christensen, K.-H. Frosch, M. Krause, M. J. Hartel

**Affiliations:** 1 Department of Trauma Surgery, Orthopaedics and Sportsorthopedics, Asklepios St. Georg, Hamburg, Germany; 2 Department of Trauma and Orthopaedic Surgery, University Medical Center Hamburg-Eppendorf, Hamburg, Germany; 3 Department of Anatomy, Christian-Albrechts-University, Kiel, Germany; 4 Department of Orthopedics and Traumatology, Cantonal Hospital Fribourg, Fribourg, Switzerland; 5 Department of Trauma Surgery, Orthopaedics and Sports Traumatology, BG Hospital Hamburg, Hamburg, Germany; 6 Department of Orthopedics and Traumatology, University Medical Center Schleswig-Holstein, Campus Kiel, Kiel, Germany

**Keywords:** Tibia, Fracture, Nanoscope, Prone, Supine, Posterolateral approach

## Abstract

**Introduction:**

The objective of this investigation was to compare different techniques to improve visualization and reduction in tibial plateau fractures involving the central lateral segments.

**Methods:**

Matched pairs of pre-fractured cadaveric tibial plateau fractures that include the central lateral segments were treated by either an anterolateral approach (supine) or PL approach (prone). Reduction was stepwise extended by additional fracturoscopy (FS), nanoscopy (NS) and lastly by epicondyle osteotomy (ECO). Reduction was analyzed by 3D scan and visualization of the lateral plateau was quantified.

**Results:**

Ten specimens (3 pairs 41B3.1, 2 pairs 41C3.3) were analyzed. Fracture steps involving the antero-latero-central (ALC) segment were insufficiently reduced after fluoroscopy using both approaches (AL 2.2 ± 1.2 mm vs PL 2.2 ± 1.0 mm, *p* 0.95). Additional NS and ECO achieved optimized fracture reduction in the ALC segment (NS AL 1.6 ± 1.3 mm vs PL 0.8 ± 0.9 mm, *p* 0.32). NS provided visualization of the entire lateral plateau (PL 102.9% ± 7.4, AL 108.8 ± 19.2%), while fracturoscopy only allowed visualization of the ALL segment and partially of PLL and ALC segments (PL 22.0 ± 23.4%, AL 29.7 ± 18.3%).

**Conclusion:**

Optimized reduction of tibial head fractures with involvement of latero-central segments requires additional video-assisted reduction or extended approaches. Nanoscopy helps visualizing of the entire lateral plateau, when compared to fracturoscopy and may become a valuable reduction aid.

## Introduction

Recent literature has focused on new classification systems that help surgeons with the decision-making process of choosing the appropriate surgical approach and intraoperative evaluation of the reduction quality [1, 2]. The 10-segment classification system as introduced by Krause et al. has been utilized in anatomical studies to evaluate the visualization of the different segments via various surgical approaches [3]. The classic anterior-lateral approach allows to reach only 36.6% of the anterior-lateral as well as the lateral articular surface but has very limited in visualization of the central lateral segments [3]. In this regard, type 41-B3 and 41-C3 fractures according to AO/OTA (Arbeitsgemeinschaft für Osteosynthesefragen/Orthopaedic Trauma Association) classification [4] are most common and difficult to treat as they often evolve the central lateral segments, namely the postero-latero-central (PLC) and antero-latero-central (ALC) segment [1, 5]. Meulenkamp et al. reported about a malreduction rate of 16.6 and 41.4% in tibial plateau fractures that were approached via an anterolateral approach and submeniscal arthrotomy or fluoroscopy alone [6]. These malreductions were heavily weighted to the posterior half of the ALC and whole PLC segment. An additional osteotomy of the lateral femoral epicondyle may be carried out in order to visualize almost all of the lateral joint line (more than 80%) [3, 7–9]. However, the modified posterolateral approach provides access to the posterior convex aspect of the articular surface (19.0% [3]), but visualization of the anterior aspect of the PLC and ALC remains limited [3]. The importance of an accurate reduction of the articular surface has been highlighted by clinical and biomechanical studies that demonstrated poorer clinical results and increased joint contact pressure if the intraarticular joint irregularity was higher than 2 mm [5, 10, 11].

Suboptimal intraoperative visualization has been described to be a main reason for inferior results [6, 12]. Fracturoscopy has been reported to be superior to fluoroscopy [12], but visualization of the central lateral segments can still be challenging.

The main scope of this study was a validation of additional methods to improve visualization in tibial plateau fractures involving the central lateral segments. Fractures were treated by an anterolateral or modified posterior-lateral approach and reduced in a “stepwise approach” by improving the visualization by (1) the additional usage of a standard 4 mm, 30-degree-angled arthroscope, (2) a 1.9-mm straight nanoscope and (3) lateral epicondyle osteotomy. We hypothesize that the AL and PL approaches both fail to properly reduce the ALC and PLC/ALC intersegmental area. We further hypothesize that additional visualization aids are necessary in this type of fractures and that by improved visualization the reduction quality of the central lateral segment can be optimized.

## Materials and methods

### Study patients

A total of ten pre-fractured specimens (*n* = 10) with the soft tissues left intact (Rimasys GmbH, Cologne, Germany) were included in this study. Using the 10-segment classification, an involvement of one or both latero-central segments (ALC and ALC/PLC intersegmental area) was defined as inclusion criteria. Fractures were classified by two independent senior orthopaedic residents according to AO/OTA and 10-segment classification [1, 4]. Two pre-fractured specimens were matched resulting in five matched pairs according to AO/OTA classification (3 41B3.1, 2 41C3.3 pairs). The PLC and ALC segments were included in all 10 specimens. Involvement of the medial segments was neglected in this study, as this was not the scope of this study. In mean, the specimens were aged 65 ± 8.4 years. Approval of the local institutional ethics committee was obtained in advance.

### Study plan

**Fig. 1:**
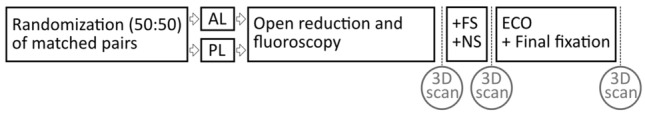
Flowchart of the fracture reduction and fixation. Matched pairs of pre-fractured specimens were randomized to be approached by either an anterolateral (AL) or posterolateral (PL) approach. Following open reduction and satisfying fluoroscopic control, a concealed 3D scan was recorded. Afterwards, fracturoscopy (FS) and nanoscopy (NS) were utilized to optimize the fracture reduction. Afterwards, an additional concealed 3D scan was recorded. Finally, an epicondyle osteotomy (ECO) was performed to further optimize the fracture reduction and final fixation was performed. In the end, another 3D scan was recorded

An overview of the methodological setup is provided in Fig. 1. A coin was flipped for a 50:50 randomization of the matched pairs and treating team: the specimens were either operated using an anterolateral approach in supine positioning or a modified posterolateral approach in prone positioning. Surgery was performed by an experienced senior surgeon (MJH, MK, MB) and assisted by an orthopaedic fellow (PB, JC, JD, GT). The proximal femur shafts were secured to the table using a screw clamp allowing for 30° rotation. Standard reduction techniques were applied and provisional fixation achieved using K-wires. The results were controlled using standard fluoroscopy (Cios Spin, Siemens, Germany) until the investigators were satisfied with the results. In the next step, a 3D scan (Cios Spin, Siemens, Germany) was performed. This scan was not viewed by the investigating team during the procedures. The scans were used for later assessment of the reduction quality. In the next step, reduction was controlled by fracturoscopy (FS) with a 4.0 mm, 30 degree angled arthroscope (Arthrex Inc.) or a 1.9 mm straight nanoscope (NS, Arthrex Inc.). Under video-assisted guidance, the reduction was optimized and temporarily fixed. Using a depth gauge, the access and visualization of the lateral plateau was quantified for both techniques. Therefore, the depth gauge was introduced from anterolateral and in cases of a modified posterolateral approach additionally from posterolateral. Another concealed 3D scan was performed following optimized reduction. In the last step, an epicondyle osteotomy (ECO) was carried out, and the reduction was further optimized if necessary. In the end, final fixation was performed, and a 3D scan recorded.

### Surgical technique

In half of the specimens, the classic anterolateral standard approach was performed in supine position [13]. Intraarticular visualization was acquired after the release of the meniscotibial ligament in the anterolateral quadrant. Via this submeniscal space, the FS and NS were inserted. Prone positioning was used for the modified posterolateral approach [7]. The posterior meniscotibial ligament and popliteomeniscal fasciculi needed to be dissected in order to reach the posterior rim of the tibial plateau. Preparation of the anterolateral window provided identical access as the classic anterolateral approach. Medial plateau fractures, if present, were left untreated in this study.

Temporary fixation was achieved using k-wires, reduction clamps and screws whenever feasible. Femoral insertion of the lateral collateral ligament (LCL) and popliteus tendons were included in the lateral epicondyle osteotomies [9, 14]. A subluxation of the meniscus was performed if necessary to gain optimal visualization of the lateral plateau. Final fixation was performed by lag screws, subcortical screws in jail technique and an anterolateral and posterolateral buttress plate (PEEKPower™ plate, Arthrex Inc.). In the end, the ECO was reduced and fixated using two cancellous screws.

### Postoperative analysis

Data of 3D scans were pseudonymized and evaluated by a senior orthopaedic trauma resident. Reduction quality was assessed based on multiplanar reconstructed scans following different steps of reduction. Fracture steps of the articular surface as well as fracture gaps were analyzed at PLC/ALC intersegmental area and within the ALC segment. Medial-to-lateral width of the tibial plateau was measured after each reduction step at its widest diameter. Based on the axial reconstruction of the lastly recorded 3D scan, the total area of the lateral plateau was measured. Proportional visualization of the lateral plateau by FS and NS was then quantified based on the measurements using a depth gauge during surgery. Proportional visualization of the lateral plateau was based on axial 3D scans at the final step of reduction and fixation.

### Statistical analysis

Data are presented as means and standard deviations (SD). The calculation was based on two groups: (1) anterolateral approach; (2) posterolateral approach. Differences between the groups were calculated with an unpaired *t *test following normality testing by Kolmogorov–Smirnov test and the Wilcoxon rank test for non-parametric parameters. Analysis was performed using GraphPad Prism 8 (San Diego, CA, USA). A *p* value < 0.05 was considered significant.

## Results

### Visualization via FS and NS (Fig. 2)

**Fig. 2:**
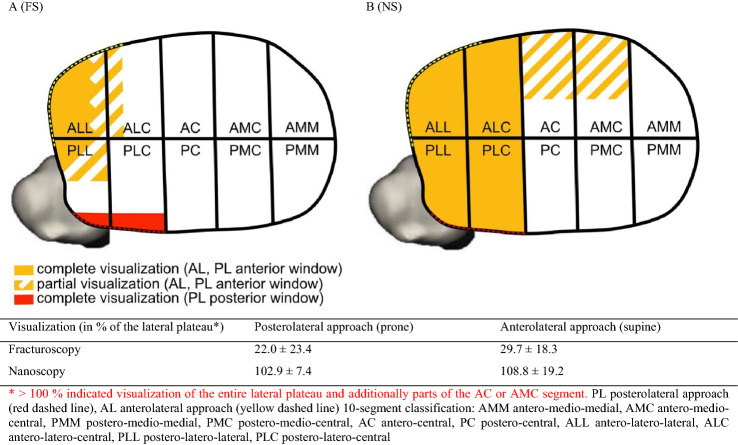
Tibial plateau visualization by fracturoscopy (FS) and nanoscopy (NS)

FS enabled visualization of the ALC and partially (criss-cross yellow lines) of the ALC and PLL resulting in 22.0 ± 23.4% of the total lateral plateau using the PL approach versus 29.7 ± 18.3% using the AL approach (*p* = 0.57). In comparison with the AL, when using the PL approach the posterior convex aspect of the PLC and PLL segment could be visualized (red). In comparison, NS significantly improved visualization of the entire lateral plateau (100%) and partially (criss-cross yellow lines) enabled insight in the antero-central (AC) and antero-medio-central (AMC) in some cases resulting in > 100% when referring to the lateral plateau as 100% (AL + FS: 29.7 + 18.3 vs. AL + NS: 108.8 ± 19.2, *p* = 0.0002 and PL + FS: 22.0 + 23.4 vs. PL + NS: 102.9 ± 7.4, *p* < 0.0001).

### Stepwise change of radiographical reduction accuracy (Table 1)

**Table 1:**
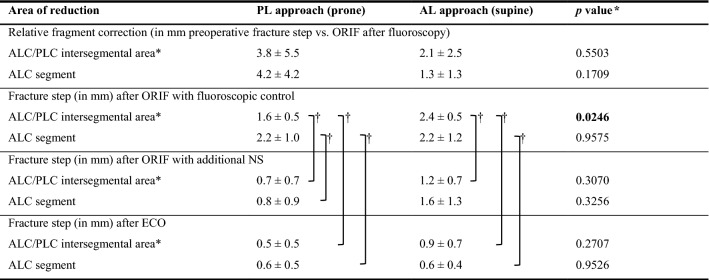
Pre-fractured specimens: reduction with or without additional FS/NS or ECO

*PL* posterolateral approach, *AL* anterolateral approach, *NS* nanoscopy, *FS* fracturoscopy, *ECO* epicondyle osteotomy, *ORIF* open reduction and internal fixation; 10-segment classification: *ALC* antero-latero-central, *PLC* postero-latero-central

*Intergroup comparison (AL vs. PL); ^†^significant difference (*p* < 0.05) within the AL and PL group, respectively

**Fig. 3:**
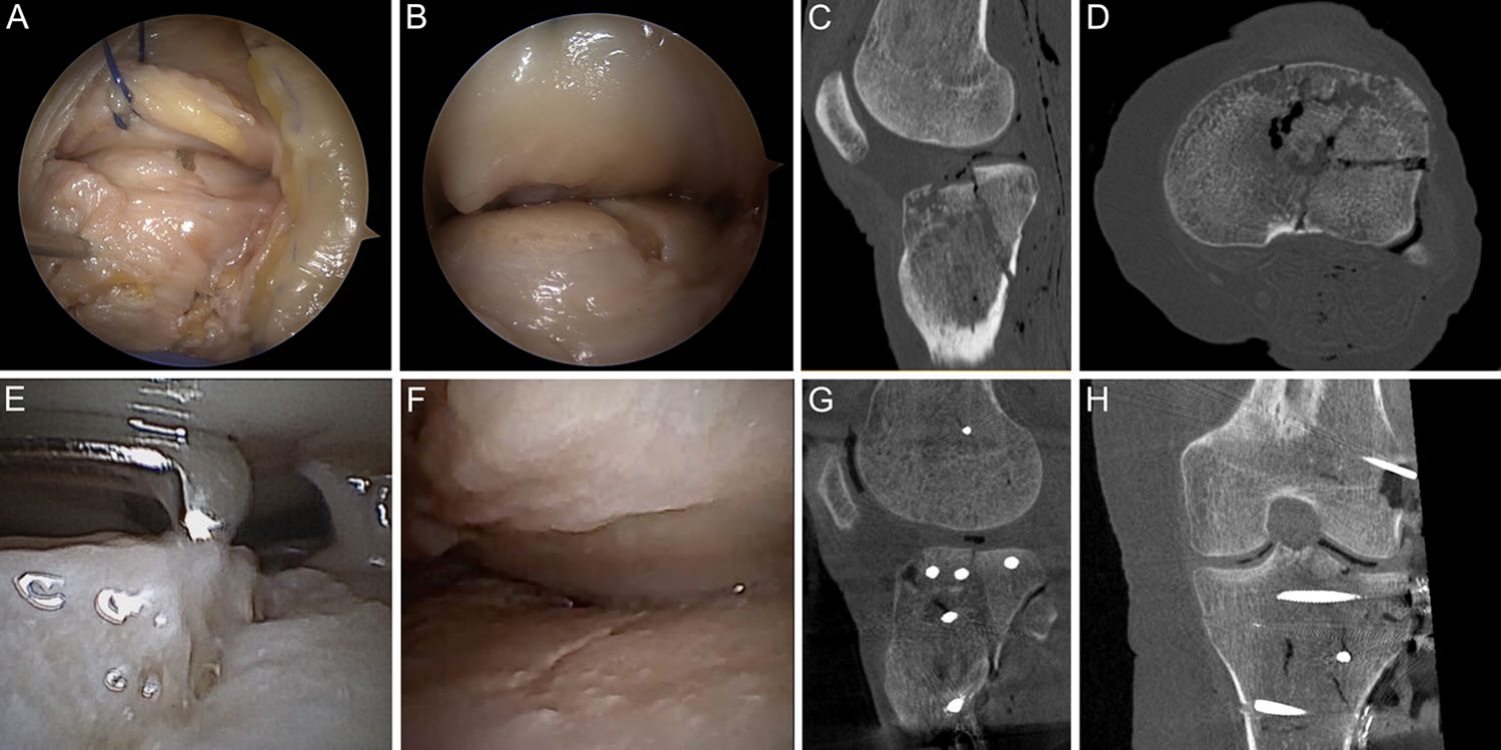
Reduction control by fracturoscopy and nanoscopy. Fracturoscopy (**A**, **B**) provides visual fracture reduction control of the peripheral aspect of the antero-latero-lateral (ALL) segment, while with nanoscopy (**E**, **F**) the fracture can be visualized even in a narrow joint compartment up to the antero-latero-central and postero-latero-central segment. Thereby, fractures involving the ALC/PLC segment (**C**, **D**) can be visualized and reduction can be optimized under visual control (**F**–**H**). An epicondyle osteotomy (**H**) was only performed due to the study protocol, but no further reduction was necessary in this case

Following open reduction and internal fixation (ORIF) and fluoroscopic-guided control, articular surface irregularity ≥ 2 mm was remaining in the ALC segment using both approaches without a significant difference. Within the intersegmental area, PL achieved significantly better reduction < 2 mm compared to the AL. No change was made using FS, but in four of five cases NS was effective in controlling an improved reduction using the AL or PL, respectively. In one case, respectively, no correction was possible due to deep impaction of the ALC fragment. Following video-assisted reduction control (Fig. 3), overall reduction quality was < 2 mm in all segments. Finally, ECO achieved fracture reduction < 1 mm in all segments, which was significantly (*p* < 0.05) different to the first reduction step using fluoroscopy. Using the AL, there a larger fracture step remained after ECO within the posterior aspect of the PLC compared to the PL (AL 1.2 ± 1.6 mm vs PL 0.2 ± 0.3 mm, *p* = 0.1994).

## Discussion

The main result of this study was that neither an anterolateral nor the modified posterolateral approach were able to control the reduction of the ALC and ALC/PLC intersegmental area with the sole use of fluoroscopy. Video-assisted reduction significantly improved the reduction within this area resulting in less than 2 mm irregularity of the articular surface. In this regard, nanoscopy was more effective as visualization of the entire plateau was possible regardless of the approach used. ECO achieved optimal reduction of the latero-central segments in all cases, but only allows limited possibility for direct reduction in the posterior half of the PLC when performed as an AL approach.

Optimal surgical reduction of tibial plateau fractures demands a strategy that takes both visualization and accessibility for direct or indirect reduction into account. Direct visualization of the articular surface has been described as a perquisite for anatomical reduction, particularly in fractures including the latero-central segments [3, 6]. By using the classic anterolateral approach only, the anterolateral third of the articular surface can be exposed [3]. In line with this, we were not able to properly reduce the ALC and ALC/PLC intersegmental area in this study following fluoroscopic control of reduction. This finding was described preciously in a clinical study, in which postoperative malreductions were heavily weighted to the posterior half of the ALC and PLC segment when using an AL approach and fluoroscopic control [6]. The PL approach yielded slightly better reduction of the ALC/PLC intersegmental area, which certainly demands on the fracture morphology of the PLC fragment. If the fragment is angulated posteriorly and exceeds far posterior in the PLC segment, it can be controlled better by the posterior window of the PL approach [15]. However, as mentioned by others, fluoroscopy could not overcome the issue of insufficient visualization in our study [6, 12, 16, 17]. Alternatively, intraoperative 3D imaging can be utilized, but it only offers a sort of snapshot in time, that cannot be used during active reduction [17]. As this study was designed as a proof-of-concept study, we aimed for anatomical reduction, while aware, that steps or gaps smaller than 2 mm may likely not be clinically relevant [5, 6]. A recent biomechanical study, again, corroborated the importance of proper fracture reduction demonstrating that a fracture step-off of 2 mm increases the joint pressure [11]. The importance of insufficient fracture reduction was shown in an analysis that evaluated postoperative CT scans, in which the authors demonstrated that insufficient intraoperative visualization may be a major reason of failure in complex tibial plateau fractures [6].

Improved visualization using standard approaches can be optimized using video-assistance and extension of the surgical approach. Fracturoscopy has shown to be superior to fluoroscopy before, leading to good clinical results [12, 18]. Depending on the patient’s knee laxity, lateral joint space opening can be 2–4 mm and atraumatic insertion of a 4-mm arthroscope can be challenging if the anterolateral wall is reduced properly. In addition, the opportunity of lateral joint space opening by distinctive varus stress is limited in bicondylar fractures and the 30-degree angulation can further limit visualization. In this study, a 1.9-mm straight nanoscope facilitated visualization of the entire tibia plateau resulting in satisfactory reduction of the remaining ALC and intersegmental area for both approaches used in this study. NS provided a better overview of the joint. All investigators felt that the handling of NS was easier and more convenient, likely due to its straight view (0° degree angulation, compared to the 30° of a standard arthroscope). Similar findings were described for visualization of small joint compartments in the ankle joint when using the nanoscope [19, 20]. NS-assisted fracture reduction may decrease the necessity of extended approaches in certain cases of complex tibia plateau fractures. Importantly, video-assistance only allows for indirect reduction of punch-like depressed fragments and the degree of preoperative fragment depression may also influence successful reduction using this technique as indicated by Krause et al. [12]. The authors suggest to critically analyze the fracture morphology of the fragment located in the ALC and ALC/PLC intersegmental area. Isolated ALC fragments with a deep preoperative depression and angulation, particularly 90° deformities, are hard to reduce indirectly and may necessitate an extension of the approach to directly reduce the fragment [21, 22]. Dislocated fragments that reach the posterior aspect of the PLC may necessitate a PL approach, which can be performed in prone or lateral position depending on the involvement of the medial plateau [15]. In fractures that only involve the anterior part of the PLC, an AL approach should be favored to spare the need for neurolysis of the peroneal nerve, which is recommended using the PL approach. Distal extension of the approach to reduce distal metaphyseal fractures is more feasible to handle using the anterolateral approach, while care must be taken to distally extend the posterior window of the posterolateral approach due to the trifurcation of the popliteal vessels.

Alternatively, to video-assisted reduction, visualization of the lateral plateau can be improved to 65% (with an isolated osteotomy of the LCL footprint at the distal femur) and 80% (with an osteotomy of both, the LCL and popliteus tendon origins) by an ECO [3, 9]. ECO in this study enabled anatomical reduction in all types of fractures, which significantly optimized the radiographic reduction quality compared to the fluoroscopic-controlled reduction. However, due to limited accessibility to the PLC and PLL segments when using the AL, larger remaining irregularities were noted at the fracture sites when compared to the PL approach. Similar findings were reported in a cadaveric and a clinical study that examined the radiological and clinical outcome of complex tibial plateau fractures [15, 23]. The authors acknowledge that the ECO procedure is an invasive procedure and that only short-term clinical results have been described for ECO in tibial plateau fractures [24]. Therefore, we emphasize the concept of a stepwise intraoperative extension allowing for superior visualization of the joint, if no sufficient reduction can be achieved using other reduction tools [8].

Clinical studies are needed to validate this proof-of-concept study design. Limitations of this study are the small number of cases, the multi-surgeon design, and a preoperatively fixed reduction sequence. Due to the fact that the sequence of the steps of the surgery was not randomized, the surgeon had the chance to improve the primary result considering a learning curve in each specimen. This may have biased the results in favor of the fracturoscopy/nanoscopy. In addition, the effect of the ECO may have been overestimated due to the reduction in the previous steps. Although matched fractures were used in this study, there are several factors regarding the fracture morphology that influence the reduction quality. Importantly, the severity of impaction and angulation are critical as well as the involvement of the medial plateau. Reduction and fixation of the medial plateau were neglected in this study, but certainly have a major impact on the surgical treatment strategy and patient positioning. However, comminuted lateral plateau fractures often coincide with split fracture types of the medial compartment, that needs separate attention using a medial approach [25, 26]. With respect to video-assisted reduction, the authors acknowledge that bleeding was absent in this study and frequently limits its application. Osteotomy of the lateral epicondyle requires a solid bone stock and should be performed restrainedly in older patients with poor bone quality. Otherwise, if the screw fixation or bone healing fails, these patients may require a constrained knee prothesis.

## Conclusion

Tibial plateau fractures involving the lateral-central segments necessitate additional aids to improve visualization. In indirectly reduceable fragments, video-assisted visualization can help to yield sufficient reduction. Here, nanoscopy provides superior visualization of the entire lateral plateau compared to fracturoscopy. Impacted isolated ALC fragments require an extension of the used surgical approach, which can be performed as extended anterolateral or extended posterolateral approach.

## Data Availability

The datasets generated during and/or analysed during the current study are available from the corresponding author on reasonable request.
